# A systems model of SDG target influence on the 2030 Agenda for Sustainable Development

**DOI:** 10.1007/s11625-021-01040-8

**Published:** 2021-10-11

**Authors:** Carl C. Anderson, Manfred Denich, Anne Warchold, Jürgen P. Kropp, Prajal Pradhan

**Affiliations:** 1 Center for Development Research (ZEF), 53115 Bonn, Germany; 2 School of Interdisciplinary Studies, University of Glasgow, Dumfries, Scotland, DG1 4ZL UK; 3 Potsdam Institute for Climate Impact Research (PIK), Member of the Leibniz Association, P.O. Box 60 12 03, 14412 Potsdam, Germany; 4 Institute for Environmental Science and Geography, University of Potsdam, Potsdam, Germany

**Keywords:** Sustainable Development Goals, Agenda 2030, Synergy, Trade-off, iMODELER, Systems model

## Abstract

The Sustainable Development Goals (SDGs) were adopted by the United Nations in 2015 as part of the “2030 Agenda for Sustainable Development” and aim to address issues ranging from poverty and economic growth to climate change. Efforts to tackle one issue can support or hinder progress towards others, often with complex systemic interactions. Thus, each of the SDGs and their corresponding targets may contribute as levers or hurdles towards achieving other SDGs and targets. Based on SDG indicator data, we create a systems model considering influence among the SDGs and their targets. Once assessed within a system, we find that more SDGs and their corresponding targets act as levers towards achieving other goals and targets rather than as hurdles. In particular, efforts towards SDGs 5 (*Gender Equality*) and 17 (*Partnerships for the Goals*) may accelerate progress, while SDGs 10 (*Reduced Inequalities*) and 16 (*Peace, Justice and Strong Institutions*) are shown to create potential hurdles. The model results can be used to help promote supportive interactions and overcome hindering ones in the long term.

**Supplementary Information:**

The online version contains supplementary material available at 10.1007/s11625-021-01040-8.

## Introduction

In 2019, the United Nations Summit on the Sustainable Development Goals (SDGs) adopted the political declaration—“Gearing up for a decade of action and delivery for sustainable development”. The remaining two thirds of the allotted 15 years must be properly utilized based on concrete actions to attain the 2030 Agenda for Sustainable Development, consisting of 17 goals and 169 targets. Achieving the SDGs will lead to “*a better and more sustainable world for all”* (United Nations 2015)*.* However, to meet all goals and targets by 2030, the actions taken to make progress towards one goal or target should not detract from the progress towards others. Instead, these actions should be mutually reinforcing or at least be neutral. Both synergies and trade-offs occur among the goals and targets within current development pathways (Lusseau and Mancini 2019; Nilsson et al. 2016; Pradhan et al. 2017; Sachs et al. 2019b).

Recently, much research has been carried out to understand interactions among SDGs that generate synergies and trade-offs (Bennich et al. 2020; Bukachi and Pakenham-Walsh 2007; Lusseau and Mancini 2019; Pham-Truffert et al. 2020; Pradhan et al. 2017; Schmidt et al. 2015). These studies either investigate interactions of a specific SDG or SDG-related theme with other SDGs [e.g., climate action (Nerini et al. 2019), social goals (Scherer et al. 2018), energy (Nerini et al. 2018), marine-related targets (Singh et al. 2018), nitrogen fertilizer (Ladha et al. 2020)] or interactions among SDGs at global scale and with all available indicator data (Lusseau and Mancini 2019; Pradhan et al. 2017; Warchold et al. 2020). Methodologically, studies on SDG interactions have been carried out using qualitative frameworks (Nilsson et al. 2016; Sachs et al. 2019a), expert elicitation processes (Nerini et al. 2019), and quantitative analysis using official or other SDG indicator data (Kroll et al. 2019; Lusseau and Mancini 2019; Pedercini et al. 2019; Pradhan et al. 2017; Zhou and Moinuddin 2017). In general, greater synergies [achievements on one goal contribute towards progress on (an)other goal(s)] rather than trade-offs [achievements on one goal detract from progress on (an)other goal(s) or vice versa] among the SDGs have been reported. Synergies can be considered *levers* for successful implementation of the 2030 Agenda, while trade-offs are potential *hurdles*.

While past studies have used systems approaches to identify synergies and trade-offs, few have applied these results to capture their indirect effects on the SDG system and its objective of *a better and more sustainable world for all.* Most studies have focused on first-order effects (direct effects between two SDGs or targets) rather than second-order effects or beyond (indirect effects that include more than two SDGs or targets; e.g., the effect of one SDG target on another is mediated by a third target). Weitz et al. (2018) investigated second-order effects of SDG interactions and highlighted that priorities for implementation of the 2030 Agenda may change when considering indirect effects in the SDG system rather than only isolated first-order SDG interactions. Therefore, development of an SDG systems model that considers directional influence among targets is crucial to capture possible indirect effects and their influence in the SDG system. In this regard, Pham-Truffert et al. (2020) use network analysis to assess interactions among the SDGs based on a literature review. Neumann et al. (2018) present a qualitative systems model of SDGs. These studies do not make use of SDG indicator data to form directional connections. Moreover, the implications of determining features of systems based on network analysis alone, such as the interconnectedness and embeddedness of SDGs and corresponding targets, do not necessarily translate into identifying systemic levers and hurdles.

Here, our study fills the above-mentioned gaps by developing an SDG systems model considering directional interactions among goals and targets of the 2030 Agenda. Based on both statistical analysis and expert assessment, we apply systems modelling to understand the influence of levers and hurdles for achieving the SDGs.

## Materials and methods

**Fig. 1:**
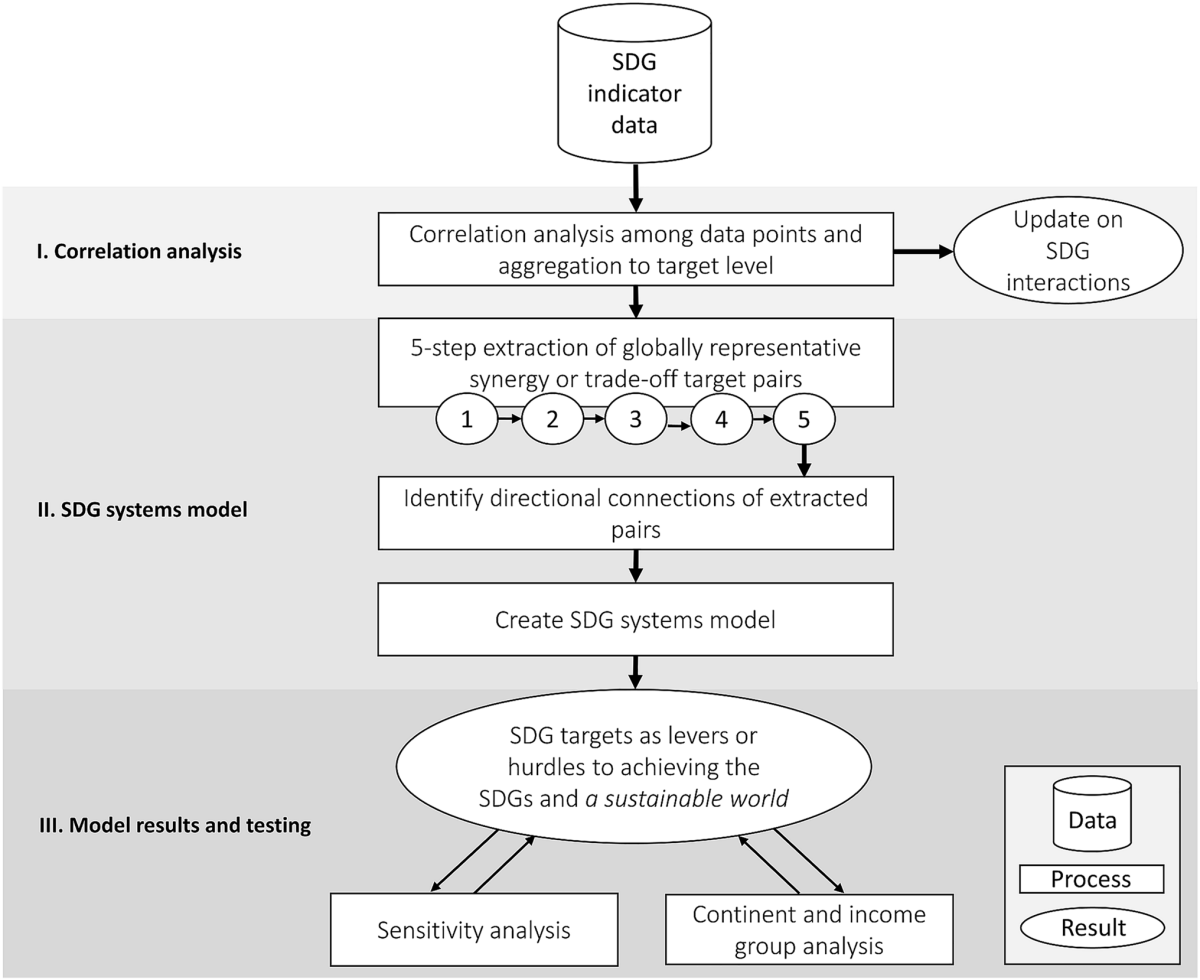
Flow diagram of analysis steps starting from Sustainable Development Goal (SDG) indicator data and ending with SDG systems model output (top to bottom). The process, with input SDG indicator data, moves from correlation analysis to SDG systems model creation and finally model testing. The “update on SDG interactions” result replicates work by Pradhan et al. (2017) in which 2016 data were used. Here, we use updated SDG indicator data through 2018

Starting with SDG indicator data from 2018 (United Nations Statistics Division 2019), a comprehensive correlation analysis is first conducted with all unique pairs of SDG indicators. Following this initial step, a series of thresholds is used to extract SDG target pairs that are both globally representative and demonstrate robust directional evidence as being either a synergy or trade-off. Afterward, the directional relations of target pairs are identified using expert knowledge. Finally, the SDG systems model is developed (Fig. 1) and sensitivity and regional (continents and income groups) analyses carried out.

### Data

We use the official SDG indicator data developed for monitoring progress towards achieving the 2030 Agenda. SDG time-series data are available for 146 of 232 indicators for a total of 251 countries and the years 1990 to 2018 (United Nations Statistics Division 2019). We include all country-disaggregated data in the analysis that are provided for 65 of the indicators in terms of population subsets, such as gender, age, urban and rural population, or income groups. The country-disaggregated data play a crucial role in monitoring progress, given the SDG motto to “leave no one behind”.

### Correlation analysis

We follow the method described by Pradhan et al. (2017) with all updated SDG indicator data from 2018 to identify interactions among the SDGs—using the nonparametric Spearman’s rank correlation (*r*_s_) analysis among pairs of data (Data S1).

Here, synergy is defined as a positive correlation between the indicator pairs with *r*_s_ value greater than 0.6 and *p* value less than 0.05. A trade-off is considered a negative correlation with *r*_s_ value less than—0.6 and *p* value less than 0.05.

The correlation analysis of indicator pairs is carried out for all 251 countries. The results of this analysis are then aggregated at the goal and target levels globally, resulting in 136 goal pairs and 5016 target pairs (Fig. 1, Table S1). The interactions of SDG indicators within and between goals or targets are quantified by their percentage of synergies, trade-offs, and non-classifieds of indicator pairs.

### SDG systems model

We build the SDG systems model based on interactions among SDG targets. Globally representative pairs of synergies and trade-offs from the correlation analysis are identified and translated into directionally influential connections. These connections are then added into an initial SDG systems model. We use the systems modelling software iMODELER (Neumann 2015) to interconnect SDG targets both within and across SDGs and determine their positive or negative influence on achieving the SDGs.

#### Extracting representative data

We apply a five-step process that extracted 131 globally representative synergies or trade-offs among the 5016 target pairs (Fig. 1, Table S1). The specific thresholds for including target pairs are determined using natural breaks in the data and setting sufficiently strict criteria to help increase the confidence of directional attribution as well as counteract differences in original SDG indicator data availability. First, we eliminate those target pairs for which either synergies or trade-offs do not describe at least 65% of the underlying indicator pairs, thereby reducing the data set by half. We refer to pairs with > 65% synergies as ‘positive’ pairs and > 65% trade-offs as ‘negative’ pairs. Second, a minimum of 15 indicator pairs is necessary, because underlying indicator data for target pairs also need to be representative. Third and fourth, only target pairs with at least four of six continents and all four World Bank Group 2018 (World Bank Group 2018) Income Groups (i.e., high, upper-middle, lower-middle, low) represented in indicators are retained, since the model should represent global trends across different continents and income groups,. Fifth, pairs of the same target are removed (e.g., Target 1.1 correlating with Target 1.1).

#### Determining direction of influence

We use expert knowledge, supported by literature and considering the description of the SDG targets (Data S2 and Table S2), to translate synergies and trade-offs into directional connections between the target pairs. The extracted 131 target pairs (97 positive pairs and 34 negative pairs) are independently reviewed by 14 experts in social and natural sciences with backgrounds in development research. Experts are asked to determine the directionality of each relation by selecting one of three options—(1) gains towards achieving the first target leads to gains towards the second, (2) vice versa, or (3) no apparent directional relation. The majority consensus among the 14 experts is selected for model inclusion. Five connections yielded tied response choices and final decisions were made after discussion among the co-authors. For target connections with a majority of ‘no apparent directional relation’ responses, target pairs are classified as ‘unexplained’. Of the 97 positive target pairs (i.e., those targets with at least 65% synergies), 66 are given a direction, while 31 are deemed ‘unexplained’, represented by divided influence between the target pair. All 34 negative target pairs (i.e., those targets with at least 65% trade-offs) were determined to be ‘unexplained’, pointing to a lack of overt explanatory evidence for translating these underlying data correlations into directional influence. ‘Unexplained’ connections are those for which there is statistical correlation, but directional influence may be indirect [e.g., increased access to energy (Target 7.1) could increase the availability of communication technology, which in turn could improve coordination of efforts to eradicate disease (Target 3.c)], or too complex at a global scale to be assigned as directional. For most SDG targets, the a priori assumption is that they will (and should) act synergistically, a position supported by intuition and more readily available evidence. Mechanisms behind the underlying negative pairs identified, on the other hand, are neither easily evidenced nor intuitive.

#### iMODELER for systems analysis

Connecting factors, synonymous with *nodes* or *variables*, in the iMODELER software implies directional influence. Directional connections alter the influence of the SDG targets that exert change within the system on other downstream targets. In addition, a multitude of reinforcing and balancing feedback loops emerge as combinations of multiple single connections and determine target influence. iMODELER is thus suitable for modeling SDG interactions given its ability to capture the impact of downstream and complex target interactions (Anderson et al. 2019; Neumann et al. 2018). Our model output is the relative influence of all targets in the SDG system on the overall achievement of the 2030 Agenda, represented by the objective *a sustainable world*. This objective reflects progress made in achieving the SDGs rather than the absolute state of arriving at a sustainable world. Nevertheless, SDGs cover reproducible human and natural capitals that are crucial for sustaining well-being over time (Arrow et al. 2012).

The influence of a target ($I_{T}$) on the objective (*a sustainable world*) is equal to the weight(s) (divided by 100) of targets and goals along the path. In the SDG systems model, with many thousands of paths, this is calculated as the aggregated sum of each individual path ($P_{i}$), where *i* goes from 1 to *n* number of paths:

$$I_{T} \, = \,\mathop \sum \limits_{i = 1}^{n} P_{i}$$

$$P\, = \, \left( {W_{S} \, \times \,\frac{{W_{1} }}{100}\, \times \,\frac{{W_{2} }}{100}\, \times \,\frac{{W_{3} }}{100}\, \times \, \cdots \, \times \,\frac{{W_{n} }}{100 }} \right)$$

The starting factor weight ($W_{S}$) is multiplied by the weights of subsequent factors along the path ($W_{n}$).

#### Model development

An initial model is first developed with all 169 SDG targets leading to their respective 17 SDGs, and then with the SDGs leading to the objective, *a sustainable world*. In the initial model, all connections from targets to goals and goals to the objective of *a sustainable world* are standardized with weights of 50 (out of 100) (Figure S1). This means each target has an influence of 25 on *a sustainable world* ($50\, \times \,\frac{50}{{100}}$) (Figure S2).

By adding connections between targets in the initial model, a new SDG systems model incorporating synergies and trade-offs is created. The model’s results are the percent changes from the initial model, i.e., increased influence (leverage potential) or decreased influence (hurdle potential) of the targets on the objective—*a sustainable world*. Because percent change is calculated, the target weights in the initial model (50) have no effect on the output—only the weights of added connections affect the output. Percent change after adding new connections is based on the initial standardized target influence of 25 (see Text S1; Figure S3 for information on model calibration).

All 131 extracted target pairs are added to the model. A target found to exert a positive influence on another target (or targets) has an increased leverage capacity towards achieving *a sustainable world*. Contrarily, a target that exhibits a negative connection broadly represents hurdle potential, since it detracts from other targets and, therefore, also from *a sustainable world* in its current systemic position. We define *levers* as those targets that, given their systemic interactions with other targets, have elevated levels of influence on achieving the SDGs as a whole. Contrarily, *hurdles* characterize those targets that require special attention and for which tackling trade-offs may be necessary regarding their contribution to achieving the SDGs.

We use weights of 25 (out of 100) for added connections in the SDG systems model, since this equates to ‘explained’ connections having twice the magnitude change in systemic influence of ‘unexplained’ connections (Text S1; Figure S3). In this way, each of the two major model inputs—the global correlation analysis and the expert assessment—contribute equally to model results. We also rerun the model with alternative added connection weights as sensitivity analyses (described in the following section).

**Fig. 2:**
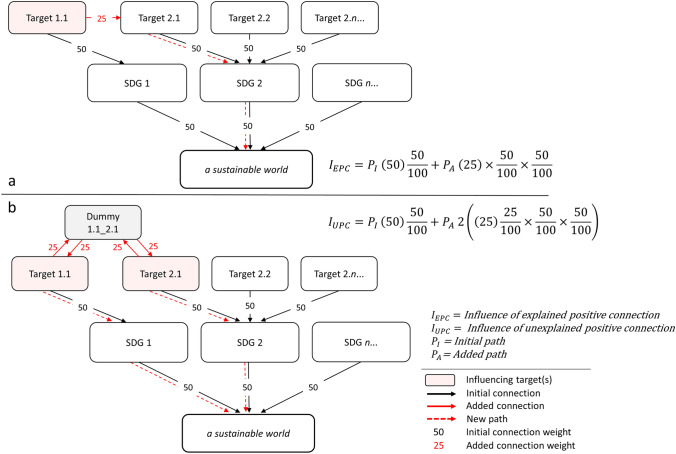
Example schematic of the process of building the Sustainable Development Goal (SDG) systems model by adding (in this case positive) ‘explained’ (**a**) and ‘unexplained’ directional connections (**b**) in iMODELER. Initial (pre-existing) connections (solid black arrows, from the initial model) link the targets to their respective goals and goals to the final factor of *a sustainable world*. When a connection is added (solid red arrows), this creates new pathways (dashed red arrows) to the subsequent SDG and *a sustainable world*. The target from which the added connection originates increases in influence by 25% for ‘explained’ connections (from 25 to 31.25 in absolute influence) (**a**) on *a sustainable world*. For new ‘unexplained’ connections (**b**), a dummy factor is created and the increase in influence for each of the two originating targets is 12.5% (from 25 to 28.125 in absolute influence)

Adding a single positive connection equates to a 25% increase in influence of the directionally exerting target from its initial value. Likewise, adding a single negative connection equates to a 25% decrease in influence. For ‘unexplained’ connections (both positive and negative), dummy factors are built in that represent the various ways in which two correlating targets could potentially be directionally connected. Each target in an ‘unexplained’ connection changes its influence by 12.5% (increase or decrease) in terms of achieving the SDGs (Fig. 2), given that influence is divided between the two pairing targets (Text S1).

Once all connections are included, complex feedback loops can emerge along pathways. iMODELER aggregates all pathways to *a sustainable world*, resulting in the changes in percent influence of all 169 targets towards this objective at both the target and aggregated goal levels.

#### Sensitivity and regional analyses

We conduct three sensitivity analyses to test the degree to which results are dependent on the application of target weights in the model (Text S2). For the first test, we create a systems model with the weights of the targets on their respective goals set to relative values based on the number of shared targets (Figure S4). A second test uses weights of 50 for all added connections. Based on model structure, this equates to an equivalent change in target strength in the system for ‘explained’ and ‘unexplained’ connections (Text S1). Using added connection weights of 10, the third test exaggerates the influence of ‘explained’ connections (± 10%) compared to ‘unexplained’ connections (± 2%). We also conduct a regional analysis to determine the relative contribution of continents and income groups to each of the top three most increasing and decreasing targets from the original global analysis.

## Results

### SDG interactions

**Fig. 3:**
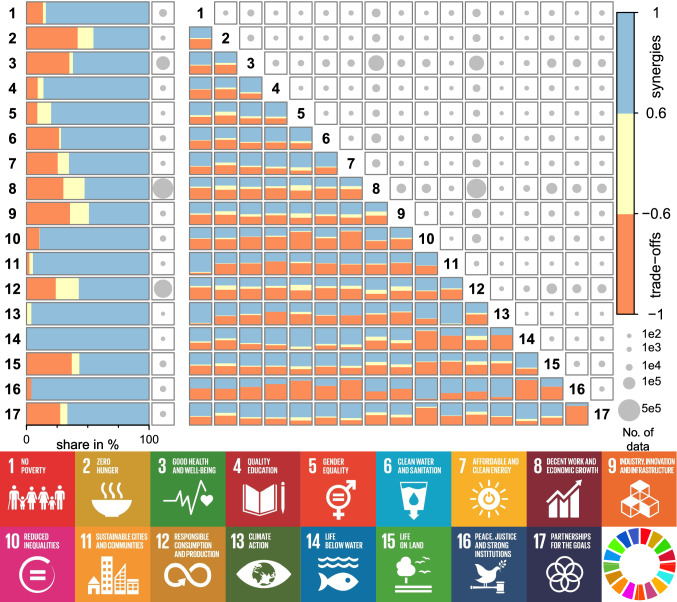
Interactions within the 17 Sustainable Development Goals [SDGs] (left) and among 136 SDG pairs (right) based on SDG data from 2018 (United Nations Statistics Division 2019). The shares of synergies (blue), non-classifieds (yellow), and trade-offs (orange) are represented by the color bars. The number of data pairs of SDG indicators is depicted by the areas of the circle in the boxes. Here, 1e2, 1e3, 1e4, 1e5, and 5e5 are 100, 1,000, 10,000, 100,000, and 500,000, respectively. The numbers and icons represent the SDGs

Based on the correlation analysis of indicator pairs, we mostly observe synergies within SDGs (i.e., among targets of the same SDG), with the share ranging from 45 to 100% of all pairs (Fig. 3, left). Interactions within SDGs 1 (*No Poverty*), 4 (*Quality Education*), 5 (*Gender Equality*), 10 (*Reduced Inequalities*), 11 (*Sustainable Cities and Communities*), 13 (*Climate Action*), 14 (*Life Below Water*) and 16 (*Peace, Justice and Strong Institutions*) result in 80–100% of the total share of synergies. For at least five of these eight goals, we observe a high share of synergies (i.e.,  ≥ 80%) for 67 countries including Argentina, Brazil, Colombia, Indonesia, Nepal, Pakistan, and Uganda (Figure S5 and Data S1). This means the achievement of a target for these goals may reinforce progress in other targets and goals in these countries. However, we also observe the share of trade-offs ranging from 30 to 42% within SDGs 2 (*End Hunger*), 3 (*Good Health and Wellbeing*), 8 (*Economic Growth*), 9 (*Industry, Innovation and Infrastructure*), and 15 (*Life on Land*). Within these five goals, we observe a high share of trade-offs (i.e., ≥ 30%) for 22 countries including Canada, China, India, Korea, and Peru (Figure S5 and Data S1). For example, our results show that an increased formalization and growth of micro-, small- and medium-sized enterprises [Target 8.3] instead of informal non-agriculture employment [Indicator 8.3.1] may impede the aim to improve resource efficiency in consumption and production [Target 8.4] by limiting material footprint [Indicator 8.4.1]. Informal activities are not necessarily more harmful to the environment than formal activities. Even on the contrary, informal economies can be more innovative, low-carbon-orientated, climate-resilient and resource-efficient than their formal counterparts (Benson et al. 2014).

Among SDGs, we identify a mostly greater than 50% share of synergies for 64 of the 136 SDG pairs and a mostly greater than 50% share of trade-offs for 38 pairs (Fig. 3, right). SDGs 1, 3, 6 (*Clean Water and Sanitation*), 7 (*Affordable and Clean Energy*) and 17 (*Partnership for the Goals*) show positive associations with 10 or more goals. For these five goals, we observe a high share of synergies (i.e., ≥ 50%) with 10 or more goals for six countries including Bolivia, China, Ethiopia, Sri Lanka, Mexico, and Mozambique (Figure S6 and Data S1). SDGs characterized by negative associations with five or more other goals include SDGs 4 (*Quality Education*), 10, 11, 12 (*Responsible Consumption and Production*), and 16. For three (SDGs 4, 10, and 16) among these five goals, we observe a high share of trade-offs (i.e., ≥ 50%) with 10 or more goals for two countries—Great Britain and South Korea (Figure S6 and Data S1).

### Levers and hurdles for achieving the SDGs

**Fig. 4:**
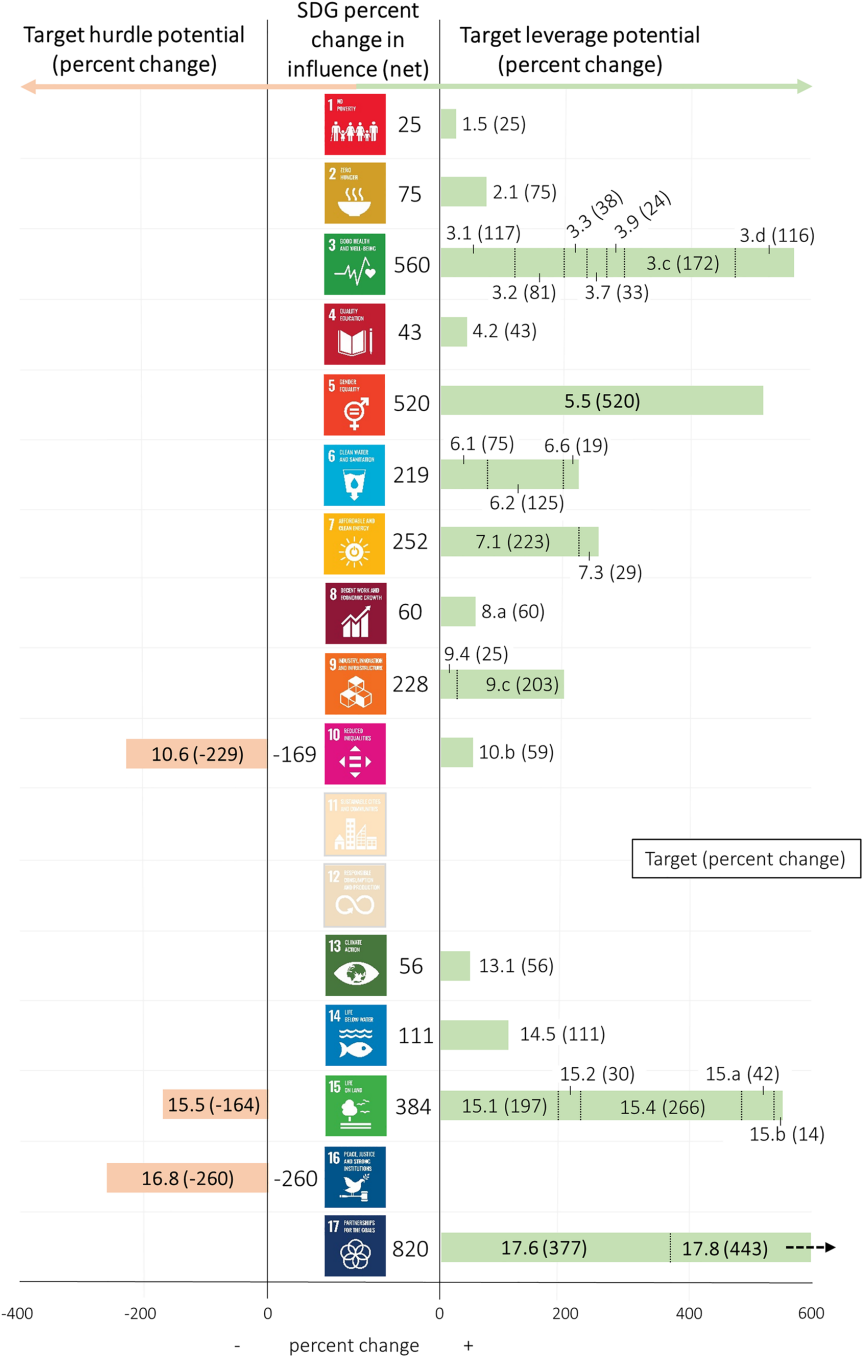
Influences of the Sustainable Development Goals (SDGs) and targets in the SDG systems model. All SDG targets that either increased or decreased compared to the initial influence on the final factor of *a sustainable world* are shown, as well as the net influence at goal level (middle). The percent increase (right) and percent decrease (left) of SDG targets represents their change in influence on the final factor due to the directional connections among targets. The three greatest increases and decreases (only three total) are in bold. SDGs 11 and 12, equally important goals for overall sustainability, had no extracted targets in the SDG systems model because of data availability and the applied exclusionary criteria (“Extracting representative data”)

Our SDG systems model includes 252 factors with 512 connections forming over 6 million feedback loops. Because all feedback loops in the model run through ‘unexplained’ connections (see Fig. 2), we do not attempt to interpret any here. Instead, our interpretation focuses on targets and goals that increase or decrease in influence on the SDG system and its objective. However, the model (and all feedback loops) can be explored in iMODELER through this read-only link: https://l.linklyhq.com/l/Kt1P. The model reveals that in total, 30 of the 169 SDG targets increase in influence on *a sustainable world* (i.e., the achievement of the SDGs as a whole), while three targets are characterized by a decrease in influence. Aggregation of target influence to goal level shows that the three most influential SDGs with leverage potential are SDGs 3, 5, and 17 (*Partnerships for the Goals*). SDGs 10 and 16 are identified as potential hurdles (Fig. 4).

#### Levers

At target level, the greatest increase in influence in the SDG system comes from Target 17.8 [strengthen the science, technology and innovation capacity for least developed countries] (+ 480%, i.e., nearly five times its original value), with Target 17.6 [knowledge sharing and cooperation for access to science, technology and innovation] (+ 384%) ranked third in this regard (Fig. 4). These results show that access to information and communication technology [Target 17.8] and multilateral North–South or South–South science and technology cooperation [Target 17.6] will have leveraging effects in the SDG system. For these targets, 34 countries have a high percentage of synergies (i.e., ≥ 80%) across more than 20 other targets, which include Brazil, France, India, Pakistan, and Vietnam (Data S1). These targets have a wide range of direct and indirect effects, mainly with targets of SDG 3 (Table S3). Other studies also highlight the important role of information and communication technology to improve health care systems (Bukachi and Pakenham-Walsh 2007; Miller and Tucker 2011) and science cooperation to address global ‘grand challenges’ which range from climate change to increasing resource depletion (Keenan et al. 2012).

Ensuring women’s full and effective participation and equal opportunities for leadership at all levels of decision-making in political, economic and public life [Target 5.5] depicts the second greatest increase in influence in our SDG systems model (+ 418%). This influence is not evenly distributed across all other SDGs, and in some cases, is indirectly affected by other SDG targets. However, in other cases the influence is significant, for example the leveraging effect of women’s participation in leadership roles [Target 5.5] on several targets within SDG 3 related to maternal [Target 3.1], neonatal, and child mortality [Target 3.2] as well as increasing the health workforce [Target 3.c] and decreasing health risk [Target 3.d]. Several studies demonstrate the potential benefits of women’s leadership in governmental organizations responsible for policies that are more supportive of women and children (Downs et al. 2014). Thirty-one countries (e.g., Cuba, Germany, Kenya, Mexico, and Poland), show a high percentage of synergies (i.e., ≥ 80%) for Target 5.5 across more than 20 other targets (Data S1). Overall, this result highlights the importance of enabling women to take up more leadership positions, as well as empowering women generally. This will likely continue to be crucial for the successful implementation of all SDGs. Emblematically, women’s empowerment will also depend on the improvement of all other SDGs.

By aggregating targets to goal level, results show that some individual targets drive a corresponding influence in their respective SDGs, while other SDGs emerge as more influential due to a greater number of less influential targets. The greatest increase in influence comes from SDG 17 (+ 865%), consisting of the second and the third most influential targets (Targets 17.6 and 17.8). SDG 3 (+ 612%) appears as the second most influential goal in the SDG system but with influence distributed among many targets (Fig. 4). SDG 3 does, however, also have strong positive connections with the top three influential targets in the entire SDG system (Targets 17.8, 17.6, and 5.5). The number of connections within SDG 3 and their varying strengths shows the importance of conducting the analysis and presenting results at target level instead of at goal level only. A target of SDG 3, eliminating preventable deaths among newborns (Target 3.2), was one of only two targets that was close to being achieved before the COVID-19 pandemic. However, the pandemic has already reversed the progress made (Pradhan et al. 2021). Therefore, lever targets with positive influences on SDG 3 will be crucial for achieving the 2030 Agenda in the post-pandemic era (e.g., Targets 17.8, 17.6, and 5.5). SDG 5 (+ 418%) is the third most influential goal, but its Target 5.5 [women’s participation in leadership and decision-making roles] has the second greatest increase in influence throughout the SDG system. Prioritizing SDG implementation requires target level analysis, since targets may not simply upscale to define the most influential goals.

#### Hurdles

The SDG systems model identifies only three SDG targets as having an overall decrease in influence towards achieving *a sustainable world* (Fig. 4). This means that these targets may create hurdles in achieving the SDGs due to their negative connections with other targets if current development processes continue. In other words, viewing these targets as embedded in a system of interacting targets, their influence on *a sustainable world* is currently less than if considered in isolation (as represented in the initial model). These targets, therefore, require special attention to reverse these trends and ensure that they are positively influencing other targets as intended for successful implementation of the 2030 Agenda.

Target 16.8 [participation of developing countries in the institutions of global governance] emerges as the greatest hurdle regarding systemic influence in the model (260%), followed by Target 10.6 (229%) [representation of developing countries in decision making in global international economic and financial institutions]. Both targets are related to developing countries and are measured with a repeating indicator—*Proportion of members and voting rights of developing countries in international organizations* [Indicators 10.6.1 and 16.8.1]. The results reflect that an increased participation of developing countries in international organizations has not and likely will not immediately lead to positive global development outcomes under the current conditions. There could be a time lag between increased participation and its actual effect on countries’ development. It is also possible that increased participation in international bodies simply has not yet translated into the adoption of sufficient policies, frameworks, and goals at the domestic level. When examining underlying trade-offs (based on negative correlations) for Targets 10.6 and 16.8, 17 countries (e.g., China, Germany, Finland, Japan, and Sweden) have a high percentage of trade-offs (i.e., ≥ 50%). These trade-offs occur across 20 other targets (Data S1). Currently, both targets [Targets 16.8 and 10.6] are negatively associated with several targets within SDG 3 related to neonatal and child mortality, epidemics, mortality due to pollution, and support for medical research [Targets 3.2, 3.3, 3.9, and 3.b].

The SDG systems model identifies the aim to protect biodiversity and natural habitats [Target 15.5] as the third greatest hurdle (164%; Fig. 4). For Target 15.5, 115 countries (e.g., Argentina, Australia, China, India, and Malaysia) have a high percentage of trade-offs (i.e., ≥ 50%) across 20 other targets (Data S1). Economic growth continues to translate into negative impacts on biodiversity, ecosystems, and environment (Sachs et al. 2019a). Currently, around one million species are at risk of extinction due to human activities (Diaz et al. 2019). In this context, our analysis shows that Target 15.5 is negatively associated with ten other targets, including four connections to SDG 3 and even two connections to targets within SDG 15 itself (see Table S3). Although we attribute all negative connections as ‘unexplained’ in the model, these connections within SDG 15 might be due to a time lag between conservation (Target 15.1 for terrestrial and inland freshwater ecosystems and Target 15.4 for mountain ecosystems) and its positive effects on reducing habitat degradation and biodiversity loss. Another possibility is that conservation efforts may be subject to a limited funding pool, thereby creating a zero-sum game for these targets. In the last decades, forest area as a proportion of total land area [Indicator 15.1.1] has decreased in 148 (out of 234) countries. At the same time, the average proportion of terrestrial and freshwater key biodiversity areas covered by protected areas [Indicator 15.1.2] has increased in 194 (out of 232) and 107 (out of 136) countries, respectively (United Nations Statistics Division 2019). Recent rates of global forest loss in protected areas have only been slightly less than those of total forest loss (Geldmann et al. 2013; Heino et al. 2015). The economic value of forested land and the over 1.1 billion people who are dependent on them for their livelihoods continue to exert pressure (Mulongoy and Babu Gidda 2008).

At goal level, the targets with the greatest decreases in influence (Targets 10.6 and 16.8) make SDGs 10 and 16 potential hurdles to attain *a sustainable world*. SDG 16 has the greatest hurdle potential among the SDGs, with no positive connections and the negative influence of only Target 16.8. Strategies towards achieving these targets and goals should be carried out with comprehensive assessments of potential negative effects on other development efforts. There is a need to uncover possible causal mechanisms that create trade-offs in relation to the indicator behind Targets 10.8 and 16.8—*changes in membership and voting rights of developing countries in international organizations*. This finding also reflects the need for making all trade-offs at target level non-obstructive so that none will be a hurdle in attaining *a sustainable world*.

### Sensitivity and regional analyses

#### Target weighting

Three models with alternate SDG target weights for added connections are created and results compared. All sensitivity analyses maintain the same top three lever targets. The first sensitivity analysis shows that among the top three lever targets, Target 17.6 moves up one rank to become the second most influential, replacing Target 5.5 which moves down to become the third most influential (Table S4). The second sensitivity analysis using equal weights for percent change in ‘explained’ and ‘unexplained’ connections shows greater variation in the magnitude of percent changes, but not in rank order. Despite equal weighting for single added connections, the number of ‘unexplained’ connections relative to ‘explained’ connections for each target does not impact the rank order of results. The third sensitivity analysis used weights of 10 for all added connections, which means ‘explained’ connections equate to five times more influence than ‘unexplained’ connections (± 10 and ± 2%, respectively). Results from this model show less variation in percent changes and closely reflect the presented results regarding rank order. The absolute percent changes in influence are shown to be highly dependent on target weights. However, the sensitivity analyses show that the rank order of SDG targets for percent increase or decrease in influence is very robust to changes in model weights (Table S4). Therefore, the validity of the ranked findings of the original SDG systems model results is supported.

#### World regions

Results of the regional analysis reveal only small variations in contributions behind the three SDG targets that increase and decrease the most in influence (Table 1). For continents, despite little variation in the most influencing targets, Europe and North America contribute slightly more and Africa contributes less to making these targets the most influential.

**Table 1:** Relative contributions of continents and incomes groups behind the three greatest lever and hurdle SDG targets (see Fig. 4). Percentages are independent of data availability, since this is controlled for

	Top 3 lever targets (percent contribution)			Top 3 hurdle targets (percent contribution)		
**Continents**	**Target 17.8**	**Target 5.5**	**Target 17.6**	**Target** **16.8**	**Target 10.6**	**Target 15.5**
Africa	12	12	13	8	8	3
Asia	19	19	18	18	18	21
Europe	20	18	17	19	19	17
North America	18	20	19	18	18	21
Oceania	15	15	16	19	19	18
South America	16	17	17	17	17	20
**Income Groups**	**Target 17.8**	**Target 5.5**	**Target 17.6**	**Target 16.8**	**Target 10.6**	**Target 15.5**
High	26	26	25	28	28	25
Upper-middle	26	25	26	25	25	26
Lower-middle	25	24	24	24	24	24
Low	23	25	24	23	23	25

*Target 17.8* strengthen the science, technology and innovation capacity for least developed countries, Target *5.5* ensure full participation in leadership and decision-making for women, *Target 17.6* knowledge sharing and cooperation for access to science, technology and innovation, *Target 16.8* strengthen the participation of developing countries in global governance, *Target 10.6* enhanced representation for developing countries in financial institutions, *Target 15.5* protect biodiversity and natural habitats

Africa plays a minor role in all top three hurdles compared to other continents. For income groups, very little variation is observed for the considered targets. Given the relatively homogenous nature of target pairs by continent and income group, the results demonstrated by the SDG systems model are supported as being accurate generalized representations of truly global trends.

## Discussion

Our study provides a novel SDG indicator data-driven operationalization of the perspective that the SDGs are a system of directionally interacting components and more than just a global collection of goals, targets, and indicators (Pradhan 2019). By creating the SDG systems model, we can observe the change in influence of all targets on the official objective of the 2030 Agenda—*a sustainable world.* This study brings several novel findings to SDG systems research.

First, an important contribution of this study is the development of a novel global SDG systems model with directional relations identified based on a data-driven approach. Going beyond second-order effects of SDG interactions (Weitz et al. 2018), our SDG systems model captures complex indirect effects among SDG connections. Besides SDGs 1 (*No Poverty*), 5 (*Gender Equality*), 6 (*Clean Water and Sanitation*) and 15 (*Life on Land*) showing mostly leverage effects across approximately 10 goals, as a result of the complex connections between targets, our model shows that SDGs 1 and 6 have relatively less influence than SDGs 5 and 15 on *a sustainable world*. The only notable similarities are for SDGs 10 (*Reduced Inequalities*) and 16 (*Peace, Justice and Strong Institutions*)—identified as the only goals with negative influence when viewed as parts of the SDG system and having negative associations (i.e., trade-offs) with 8 and 11 other goals, respectively. This new understanding of levers and hurdles based on a systems model can provide starting points for sustainable transformation. Its relevance is strengthened given the current situation in which the progress made towards the SDGs has been insufficient and the COVID-19 pandemic has regressively impacted many goals (Health 2020; Pradhan et al. 2021).

Second, this study shows that most countries share challenges and opportunities for achieving the objective of the 2030 Agenda—*a sustainable world*. Our results of the regional analysis only show small variations in contributions of the continent and income groups in the most positively or negatively influencing targets. This result is in line with Kroll et al. (2019), who show similar challenges for countries regarding SDG trade-offs and synergies across income groups, although it is in contrast to Lusseau and Mancini (2019), who find that low income countries do not exhibit antagonistic SDG targets. However, when comparing continents, efforts towards achieving the SDGs in Africa are the least likely to create potential hurdles with other SDGs. In particular, hurdles related to participation of developing countries [Targets 16.8 and 10.6] are much weaker in Africa. It can be expected that as African countries become members of international organizations there will be more concurrent gains in development than in other regions.

Third, our results emphasize the role of gender equality [SDG 5] and international cooperation [SDG 17] for achieving *a sustainable world*. We find that women’s participation in decision-making [Target 5.5] has the second greatest leverage potential. This shows that gender equality in employment and other related domains can be self-sustaining because of the positive feedback effects from gender equality to economic and social well-being—two of the three sustainability dimensions. Pham-Truffert et al. (2020) find that SDG 5 does not create any trade-offs but is not strongly synergist, while SDG 17 is a “systemic multiplier of synergies”. Our findings contrast with Warchold et al. (2021), who use disaggregated SDG indicator data correlations to show that SDGs 5 and 17 have a high number of linear trade-offs. Nevertheless, the empowerment of women and girls leading to synergistic development gains is widely supported (Lim et al. 2018; Nilsson et al. 2016; Sachs et al. 2019a; Seguino 2016). Empowerment must go beyond this paper's findings of increasing women’s participation in leadership roles and permeate societies across the world.

Although the COVID-19 pandemic has eroded progress made on gender equality and women’s empowerment, the leveraging potential of SDG 5 provides a window of an opportunity for sustainable transformation in the post-pandemic era (Nature Editorial 2020; Pradhan et al. 2021; Wenham et al. 2020). The pandemic has also underscored the importance of leadership roles for woman, since some evidence suggests that countries with women leaders have thus far had better COVID outcomes on average (Garikipati and Kambhampati 2021). Targets 17.6 (knowledge sharing and cooperation for access to science, technology and innovation) and 17.8 (strengthen the science, technology and innovation capacity for least developed countries) also act as strong levers for development gains. Responses during the pandemic, i.e., the positive health outcomes stemming from collaboration in medical research including North–South and South–South cooperation at the various levels, have also demonstrated the leveraging impacts of SDG 17 (Nerini et al. 2020). Sachs et al. 2019b propose six societal transformations for achieving the SDGs in which addressing gender inequalities, technology adoption, and economic growth are grouped within the same transformation given that such interventions are “… synergistic with no major trade-offs.” Efforts towards achieving these lever targets should consider the potentially wide-ranging progress they can actuate among other targets.

We develop the SDG systems model based on the outcomes of past systemic interactions among indicators across countries and through years aggregated at the global level. The levers and hurdles identified are thus the products of current development trends and may not persist as progress is made and SDGs are achieved. Along with critiques of bias against developing countries (Wackernagel et al. 2017), the availability and level of disaggregation of SDG indicator data also constrain the breadth and accuracy of the results. For example, SDGs 11 and 12, while important for achieving *a sustainable world,* were only represented in the correlation analysis by one and two targets, respectively. These targets did not subsequently meet the criteria for model inclusion. Targets that met all thresholds as correlating pairs that could be included in the systems model originally had an average indicator data point count of 141, whereas excluded targets had on average 128. This indicates a slight distortion in results due to data availability. However, this difference is minimal and our five-step extraction process was successful at excluding targets with many data points and only including targets with few data points on the basis of global significance. Our interpretation of directional influence from correlation must also be approached carefully. We purposefully do not claim that these connections are “causal” but rather see our methodology as an attempt at approaching causality. The iMODELER software is designed for causal interactions, since it allows the user to assign directional influence of varying weights. As the SDG indicator data improve and the body of relevant research advances, it may be possible to apply our method with very few ‘unexplained’ connections and sufficient evidence of causality. For now, our five-step data extraction process was designed using thresholds to include only substantial evidence of global trends for proper interpretation of directional influence among the targets. While most of the target pairs show very high agreement, it is possible that a different set of experts or a different approach for determining connections could influence results. However, the sensitivity analyses show that the rank order of influential targets is a robust finding and unlikely to change under these parameters. We recognize the diversity of approaches to creating SDG systems models (Bennich et al. 2020) and see our novel approach as contributing to this body of work.

## Conclusions

Although progress in isolated indicators represents progress towards achieving the SDGs de facto, sustainable development relies on mutually reinforcing gains rather than incremental steps in isolation (Pradhan 2019; Sachs et al. 2019a, b). Moreover, SDG indicators are tracked regularly but their interactions have not received enough attention. The novel SDG systems model presented here is the first version of a model for forthcoming research aimed at developing a dynamic SDG model that considers interactions among SDGs and their targets through time. Dynamic SDG models allow for different experimentations on SDG interactions and their influences towards *a sustainable world*. The results of these experiments can support decisions on prioritization of actions for SDGs, which is crucial during the ongoing COVID-19 pandemic that has halted or reversed progress made in many SDGs. Our model could contribute to the difficult process of identifying priorities among so many diverse targets in the post-pandemic era (Naidoo and Fisher 2020).

Currently, our model shows more levers than hurdles among SDG goals and targets. In addition, the analyses suggest that under current conditions, increased participation of developing countries in international organizations has not and will not immediately lead to *a better and more sustainable world for all*, i.e., the overall goal of the SDGs and the path towards it are not a priori inclusive. Further analyses with new data will have to show whether this observation, which may have serious consequences, can be maintained.

For now, several generalized policy recommendations emerge from the results. First, our findings support emphasizing gender equality and particularly having women in leadership roles (Target 5.5). Creating the necessary enabling conditions through concurrent development gains in sectors such as health (SDG 3) will likely lead to gains. As progress is made and women fill more leadership roles, it is also reasonable to expect a long term positive feedback on the development foci that supported their empowerment. Based on our findings, this recommendation applies to all regions and income groups. Second, the findings support an emphasis on global collaboration and technology exchange (Targets 17.6 and 17.8) given the many potential concurrent development gains that can arise from these efforts. Developed countries can ensure that development aid and cooperation efforts assess and capitalize on potential lever effects. For developing countries, creating institutional arrangements and intellectual capital that allow for effective collaboration may be emphasized. In contrast, third, caution is warranted globally in relation to the potential hurdles associated with gains in participation and voting rights of developing countries in international institutions (Targets 10.6 and 16.8). We must ensure that these development gains do not detract from progress towards other goals and targets, although we find this is likely a less important consideration for African countries. This finding urgently calls for further research to reveal potential causal mechanisms.

We expect that progress towards achieving the SDGs will evolve in the coming years and shifts in model results will provide further insight. In this way, such models can be used as monitoring tools not only to understand progress towards achieving the SDGs, but also to identify whether hurdles have been overcome. The methods that we have applied to develop the SDG systems model at the global scale can also be replicated at or adapted to regional, national, and local levels to assess SDG systems at various spatial scales or for specific scenarios. The reduced complexity at a lower spatial level would also improve method validation and be better positioned to explain the mechanisms behind ‘unexplained’ target and goal interactions. A further understanding of directional influence within the SDG system would require replacing ‘unexplained’ connections or the addition of complementary connections to the model. Finally, we encourage comparative studies of existing SDG systems modelling methods, given the urgency to deliver sustainable development.

## Supplementary Information

Below is the link to the electronic supplementary material.

Supplementary file including Text S1-S2; Figures S1-S4: Table S1-S4 (PDF 293 KB)

Data S1 (7Z 51140 KB)

Data/Table S2 (XLSX 47 KB)

Figure S5 (PDF 642 KB)

Figure S6 (PDF 2548 KB)
