# Supplementary material for: A systems model of SDG target influence on the 2030 Agenda for Sustainable Development
Source: Sustain Sci. 2021 Oct 11;17(4):1459–72. doi: 10.1007/s11625-021-01040-8 (PMC8504570; doi:10.1007/s11625-021-01040-8)
Supplement: Supplementary file 1 — Supplementary file including Text S1-S2; Figures S1-S4: Table S1-S4 (PDF 293 KB) [file 11625_2021_1040_MOESM1_ESM.pdf]

A systems model of SDG target influence on the 2030 Agenda for Sustainable Development

Carl Anderson [1,2], Manfred Denich [1], Anne Warchold [3], Jürgen P. Kropp [3,4], Prajal Pradhan [3,*]

[1] Center for Development Research (ZEF), D-53115 Bonn, Germany

[2] School of Interdisciplinary Studies, University of Glasgow, Dumfries, Scotland DG1 4ZL, UK

[3] Potsdam Institute for Climate Impact Research (PIK), Member of the Leibniz Association, P.O. Box 60 12 03, D-14412 Potsdam, Germany

[4] University of Potsdam, Institute for Environmental Science and Geography, Potsdam, Germany

*pradhan@pik-potsdam.de

Supplementary Data

Data S1. The folders contain data and results from the correlation analysis. Please see READ ME file for details

Data S2. Outcomes of expert assessment on directional linkages between SDG target pairs

Supplementary Text

Text S1. Sustainable Development Goal systems model calibration

The calibration of the SDG systems model is guided first by deciding on its structure (i.e., how the SDG targets and goals are connected). Therefore, we first describe the decision process behind the structure of the model before explaining the numeric calibration of target weights, goal weights and added connection weights. The latter process is guided by exploring the effects on percentage change in target influence based on a single added

connection. As described below, we aim to calibrate the model so that it reflects the input data as well as SDG systems theory.

Within the model, 131 directional connections are added. Due to the model structure, this creates complex feedback loops. These loops amplify or attenuate the influence of the SDG targets and goals within their newly formed system (based on added connections).

<u>Model structure</u>

As shown in Figure S2, the model is created first by connecting SDG targets to their corresponding goals and goals to the objective. The latter is named *a sustainable world* and represents the collective achievement of all SDGs. Based on the correlation analysis with thresholds and expert assessments, directional connections are added to the model. ‘Explained’ connections describe those for which consensus among experts resulted in a direction (i.e., gains towards achieving one target lead to gains towards another). ‘Unexplained’ connections describe those for which there is statistical evidence of a potential connection based on the global correlation analysis and threshold criteria, but a majority of experts were not able to assign a direction to the relation. To add explained and unexplained connections into the model, a representative structure is created for each. For explained connections, a new directional connection is added from the target exerting positive or negative impact on another target (see Target 1.1. in Figure 1 main text). For unexplained connections, we build in a third ‘dummy’ variable that acts to connect the targets involved in the connection. The dummy variable represents the potential indirect relation between the targets, which is based on the statistical evidence from the analysis. In this relation, e.g., Target 1.1 can exert influence on the dummy variable, which in turn exerts influence on Target 2.1, or vice versa (Figure 1 main text).

Having established the described structural relations as most closely representing the evidence for target relations based on our analysis of correlations, thresholds, and expert assessments, we then determine the weights of connections in the model.

Single connection calibration

The aim of our research is to determine the percent change in influence of SDG targets on *a sustainable world* once they are embedded within a system of all interacting SDG targets and goals. Because the output is percent change from initial influence, our results are not impacted by the weights of targets to goals and goals to *a sustainable world.* The correlation analysis and expert assessment are conducted at SDG target level, and therefore all added connections to the model are among targets. Because iMODELER calculates influence of the SDG targets based on the weights of downstream paths, and the paths from targets to goals and goals to *a sustainable* world are standardized across the model, these weights have no impact. We select weights of 50 for all connections between SDG targets and goals and connections between goals and *a sustainable world*. This equates to an initial influence of 25 for all targets on *a sustainable world.* Likewise, the influence of 25 does not impact results. Rather, percent change (increase or decrease) from an initial value of 25 is calculated.

However, the weights of *added* connections do have an impact on percent change of target influence. Therefore, we explore different weights (1-100) for these connections and determine a theoretically sound solution. Note that negative connections are governed by the same structures and formulas, only with equivalent negative starting weights (-25). Therefore, we only describe positive connections here.

Based on the model structure described above, to calculate the influence of adding one **explained** positive connection, the following formula is used:

$$I_{EPC} = I_{IT} + \left(W_N \times \frac{W_{TG}}{100} \times \frac{W_{GS}}{100}\right)$$

$$I_{EPC} = 25 + \left(W_N \times \frac{50}{100} \times \frac{50}{100}\right)$$

Based on the model structure described above, to calculate the influence of adding one **unexplained** positive connection, the following formula is used:

$$I_{UPC} = ITF + 2\left(W_N \times \frac{W_N}{100} \times \frac{W_{TG}}{100} \times \frac{W_{GS}}{100}\right)$$

Or

$$I_{UPC} = 25 + \left(W_N \times \frac{W_N}{100} \times \frac{50}{100} \times \frac{50}{100}\right)$$

I_{EPC} = Influence of explained positive connection
I_{UPC} = Influence of unexplained positive connection
I_{IT} = Initial target influence
W_N = Weight of new connection
W_{TG} = Weight of connection from SDG target to goal
W_{GS} = Weight of connection from goal to a sustainable world

The derived values are then used to calculate percent change from initial target influence. Using this formula, we graph the percent increase in target influence on *a sustainable world* based on added connection weights of 1-100 (Figure S4).

Because a feedback loop is formed in the structure of the unexplained connection, its increase is non-linear. Using added connection weight values of greater than 50, an unexplained connection has a greater increase in influence than an explained connection

in the model. At the added connection value of 50, the percent increase is equal for both explained and unexplained connections (50%), and below 50, the explained connection increases more than the unexplained connection in influence. The latter configuration aligns with the evidence, given that explained connections, which are based also on the expert assessments, should create greater percent changes in the model targets.

At a starting weight of 25, the explained connections increase 25% and the unexplained connections increase exactly half of this, 12.5%. Therefore, starting with added connections of weight 25 implies that connections determined by the expert assessment portion of the analysis are exerting twice the weight of connections based only of the correlation analysis and thresholds. Because our aim is for each primary step in model development (global correlation analysis and expert assessment of directions) to have equal influence in final results, we select this model calibration for adding individual connections.

Text S2. Sensitivity and regional analyses

<u>Test 1. Relative weights</u>

For the first test, we create a systems model with a different initial conceptualization of how the SDG targets contribute to their respective goals and, ultimately, *a sustainable world*. Here, the weights of the targets on their respective goals are set to relative values based on the number of shared targets. These relative values are standardized so that the sum of all the targets within any SDG is equal to 100 (Figure S4). Using relative weights, this model assumes that the targets' strengths in terms of achieving the SDGs are based on their contribution to their individual goals. The implication is such that if we meet, for example, five of the ten total targets for SDG 10, we have only achieved 50% of

one of seventeen SDGs. If we achieve five out of the seven targets in SDG 1 (71%), we have again achieved five targets in total, but relative weighting implies that we are closer to achieving the aggregate goal of achieving all 17 SDGs together.

Test 2. Added connection weight of 50

The second test applies weights of 50 to all added connections in the model. Due to the structure of the model, this results in equal percent changes in target influence for explained and unexplained connections (Text S1). This has the effect of creating no difference in target influence percent change occurring despite the expert assessment of direction.

Test 3. Added connection weight of 10

The third test applies weights of 10 to all added connections in the model. Due to the structure of the model, this results in percent changes of 10% in target influence for explained connections and only 2% for unexplained connections (Text S1). This test therefore increases the impact of the expert assessment portion of the methodology by making explained connections five times stronger than unexplained connections.

Regional analysis

For the regional analysis, results of the SDG systems model at global level are further explored to reveal the underlying regional contributions of the top three levers and hurdles (three targets that increase or decrease the most in influence, respectively). The SDG indicator pairs aggregated at target level are used as the starting data and grouped based on continent (i.e., Africa, Asia, Europe, North America, Oceania, and South America) as well as income group (upper, upper-middle, lower-middle, and lower). The data extraction thresholds used in the model based on continent coverage and income groupings are excluded from this analysis, with steps 1, 2, and 5 reapplied (see Table S1). Because

income group classification and continent classification are highly associated, it is important to exclude both of these steps to determine the controlled effects of each threshold on the results.

For each of the six targets (top three levers and hurdles), we count the number of positive and negative target pairs and calculate their percentage relative to total pairs. This procedure controls for data availability, since some targets may have more pairs merely as a result of greater representation in the underlying SDG indicator data. Subsequently, we sum these percentages and calculate the percentage of that sum represented by each continent or income group. This final percentage is the relative contribution of each of the regions to each of the top three most increasing and decreasing targets from the original global analysis. The percentages thus show to what degree the trends from the global analysis are truly global or instead disproportionately caused by certain continents or income groups.

Supplementary Figures

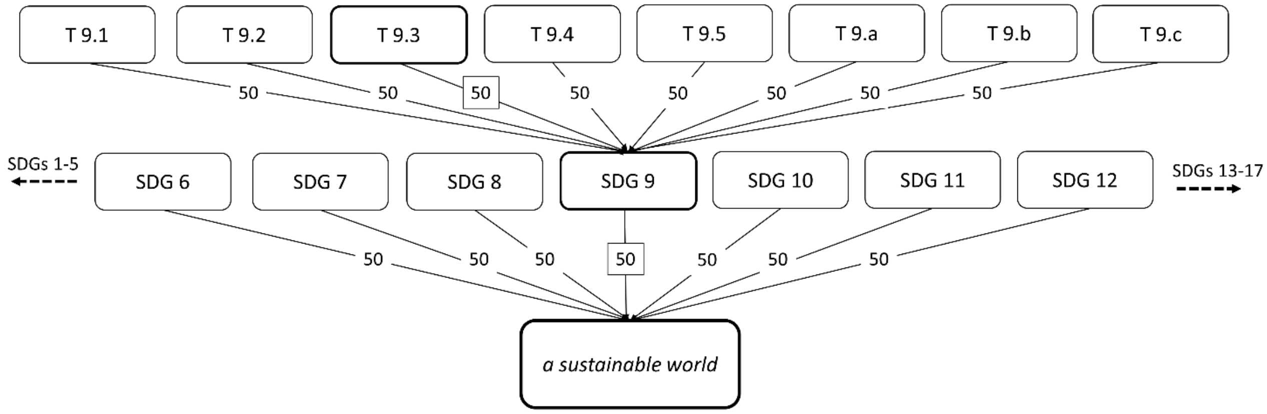

Figure S1. Initial SDG systems model structure. SDG targets (here, SDG 9 targets, e.g. T 9.3; outlined in bold) lead to SDG 9 with weights of 50, while SDG goals (e.g. SDG 9; outlined in bold) lead to *a sustainable world* also with weights of 50.

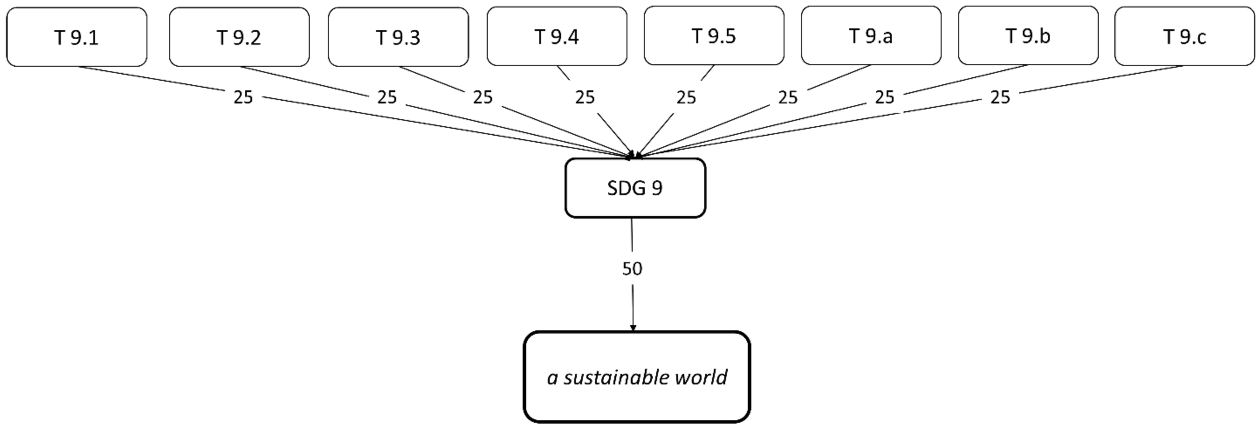

Figure S2. Influence of targets in the initial SDG model on *a sustainable world*. All SDG targets (here, SDG 9 targets) have an influence of 25 on *a sustainable world* while all SDGs (here, SDG 9) have an influence of 50.

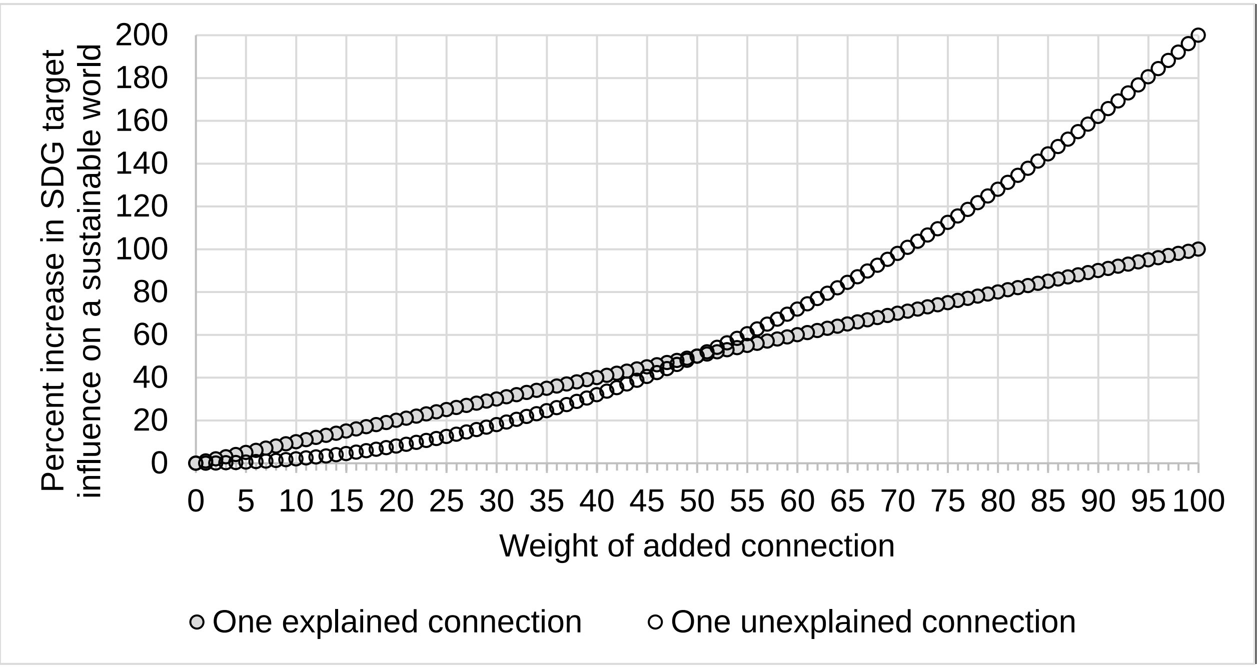

Figure S3. Percent increase in Sustainable Development Goal (SDG) target influence on *a sustainable world* when adding a single explained positive connection or a single unexplained positive connection with weights from 1 to 100.

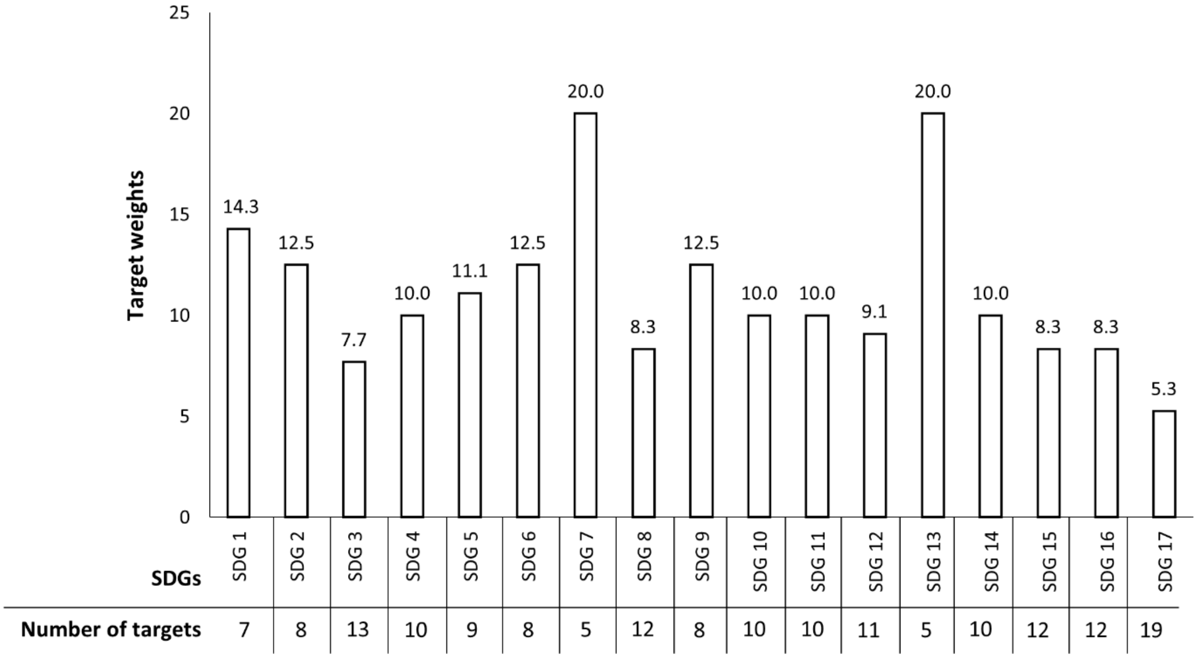

Figure S4. Relative systems model weighting for sensitivity analysis (Test 1) with the number of targets and target weights per Sustainable Development Goal (SDG). The weight of each target is determined by dividing the number of shared targets within any SDG by 100.

Figure S5. *(PDF file)* Interactions among the 136 pairs of Sustainable Development Goals [SDGs] based on SDG data from 2018 (United Nations Statistics Division, 2019) at country level, denoted by its ISO code. The shares of synergies (blue), non-classifieds (yellow), and trade-offs (orange) are represented by the color bars. The number of data pairs of SDG indicators is depicted by the areas of the circle in the boxes. The icons represent the SDGs.

Figure S6. *(PDF file)* Interactions within the 17 Sustainable Development Goals [SDGs] based on SDG data from 2018 (United Nations Statistics Division, 2019) at country level, denoted by its ISO code. The shares of synergies (blue), non-classifieds (yellow), and trade-offs (orange) are represented by the color bars. The number of data pairs of SDG indicators is depicted by the areas of the circle in the boxes. The icons represent the SDGs.

Supplementary Tables

Table S1. Five-step extraction of significant and globally representative target pairs based on underlying indicator data. This pre-processing step is depicted in Figure 1. An initial 5016 target correlation pairs were reduced to 131 based on whether indicator pairs were either 65% positive or 65% negative (1), composed of at least 15 indicator pairs (2), composed of indicator pairs from at least four of six continents (3), composed of at least one indicator pair from every income group of countries (4), and not composed of duplicate targets (5).

Step	Correlation pairs	Threshold	Remaining Correlations	% of Total
1	5016	65%	2416	48
2	2416	15 Indicator pairs	1901	38
3	1901	4 of 6 Continents	602	12
4	602	4 of 4 Income groups	285	6
5	285	Within-target interactions	131	5

Table S2. (Excel file). Empirical evidence for directional connections. We use expert knowledge to translate synergies and trade-offs into directional connections between the target pairs. This process is supplemented by an ad-hoc literature search. Lacking author agreement and empirical evidence, target pairs are classified as ‘unexplained’.

Table S3. 'Explained' and 'unexplained' connections for the top three most influential lever and hurdle targets (see Figure 3 main text).

Lever targets

(1) ***17.8*** *Fully operationalize the technology bank and science, technology and innovation capacity-building mechanism for least developed countries by 2017 and enhance the use of enabling technology, in particular information and communications technology*

'explained'

**Target 17.8 →**	Target 3.1	By 2030, reduce the global maternal mortality ratio to less than 70 per 100,000 live births
	Target 3.2	By 2030, end preventable deaths of newborns and children under 5 years of age, with all countries aiming to reduce neonatal mortality to at least as low as 12 per 1,000 live births and under-5 mortality to at least as low as 25 per 1,000 live births
	Target 3.9	By 2030, substantially reduce the number of deaths and illnesses from hazardous chemicals and air, water and soil pollution and contamination
	Target 3.d	Strengthen the capacity of all countries, in particular developing countries, for early warning, risk reduction and management of national and global health risks
	Target 6.2	By 2030, achieve access to adequate and equitable sanitation and hygiene for all and end open defecation, paying special attention to the needs of women and girls and those in vulnerable situations
	Target 7.1	By 2030, ensure universal access to affordable, reliable and modern energy services
	Target 9.c	Significantly increase access to information and communications technology and strive to provide universal and affordable access to the Internet in least developed countries by 2022
	Target 14.5	By 2020, conserve at least 10 per cent of coastal and marine areas, consistent with national and international law and based on the best available scientific information

	Target 15.1	By 2020, ensure the conservation, restoration and sustainable use of terrestrial and inland freshwater ecosystems and their services, in particular forests, wetlands, mountains and drylands, in line with obligations under international agreements
	Target 15.4	By 2030, ensure the conservation of mountain ecosystems, including their biodiversity, in order to enhance their capacity to provide benefits that are essential for sustainable development

‘unexplained’

**Target 17.8 → ? ←**	Target 5.5	Ensure women’s full and effective participation and equal opportunities for leadership at all levels of decision-making in political, economic and public life

(2) ***5.5*** *Ensure women’s full and effective participation and equal opportunities for leadership at all levels of decision-making in political, economic and public life*

‘explained’

**Target 5.5 →**	Target 3.1	By 2030, reduce the global maternal mortality ratio to less than 70 per 100,000 live births
	Target 3.2	By 2030, end preventable deaths of newborns and children under 5 years of age, with all countries aiming to reduce neonatal mortality to at least as low as 12 per 1,000 live births and under-5 mortality to at least as low as 25 per 1,000 live births
	Target 3.c	By 2030, ensure that all girls and boys have access to quality early childhood development, care and pre-primary education so that they are ready for primary education
	Target 3.d	Substantially increase health financing and the recruitment, development, training and retention of the health workforce in developing countries, especially in least developed countries and small island developing States
	Target 4.2	Strengthen the capacity of all countries, in particular developing countries, for early warning, risk reduction and management of national and global health risks

	Target 14.5	By 2020, conserve at least 10 per cent of coastal and marine areas, consistent with national and international law and based on the best available scientific information
	Target 15.1	By 2020, ensure the conservation, restoration and sustainable use of terrestrial and inland freshwater ecosystems and their services, in particular forests, wetlands, mountains and drylands, in line with obligations under international agreements
	Target 15.4	By 2030, ensure the conservation of mountain ecosystems, including their biodiversity, in order to enhance their capacity to provide benefits that are essential for sustainable development

'unexplained'

**Target 5.5 → ? ←**	Target 7.1	By 2030, ensure universal access to affordable, reliable and modern energy services

(3) ***17.6*** *Enhance North-South, South-South and triangular regional and international cooperation on and access to science, technology and innovation and enhance knowledge-sharing on mutually agreed terms, including through improved coordination among existing mechanisms, in particular at the United Nations level, and through a global technology facilitation mechanism*

'explained'

**Target 17.6 →**	Target 2.1	By 2030, end hunger and ensure access by all people, in particular the poor and people in vulnerable situations, including infants, to safe, nutritious and sufficient food all year round
	Target 3.1	By 2030, reduce the global maternal mortality ratio to less than 70 per 100,000 live births
	Target 3.2	By 2030, end preventable deaths of newborns and children under 5 years of age, with all countries aiming to reduce neonatal mortality to at least as low as 12 per 1,000 live births and under-5 mortality to at least as low as 25 per 1,000 live births

	Target 7.1	By 2030, ensure universal access to affordable, reliable and modern energy services
	Target 14.5	By 2020, conserve at least 10 per cent of coastal and marine areas, consistent with national and international law and based on the best available scientific information
	Target 15.4	By 2030, ensure the conservation of mountain ecosystems, including their biodiversity, in order to enhance their capacity to provide benefits that are essential for sustainable development
	Target 17.8	Fully operationalize the technology bank and science, technology and innovation

Hurdle targets

(1) ***16.8*** *Broaden and strengthen the participation of developing countries in the institutions of global governance*

'unexplained'

**Target 16.8 → ? ←**	Target 3.2	By 2030, end preventable deaths of newborns and children under 5 years of age, with all countries aiming to reduce neonatal mortality to at least as low as 12 per 1,000 live births and under-5 mortality to at least as low as 25 per 1,000 live births
	Target 3.3	By 2030, end the epidemics of AIDS, tuberculosis, malaria and neglected tropical diseases and combat hepatitis, water-borne diseases and other communicable diseases
	Target 3.9	By 2030, substantially reduce the number of deaths and illnesses from hazardous chemicals and air, water and soil pollution and contamination
	Target 3.b	Support the research and development of vaccines and medicines for the communicable and non-communicable diseases that primarily affect developing countries, provide access to affordable essential medicines and vaccines, in accordance with the Doha Declaration on the TRIPS Agreement and Public Health, which affirms the right of developing countries to use to the full the provisions in the Agreement on Trade-Related Aspects of Intellectual Property Rights regarding flexibilities to

		protect public health, and, in particular, provide access to medicines for all
	Target 5.5	Ensure women's full and effective participation and equal opportunities for leadership at all levels of decision-making in political, economic and public life
	Target 7.1	By 2030, ensure universal access to affordable, reliable and modern energy services
	Target 10.b	Encourage official development assistance and financial flows, including foreign direct investment, to States where the need is greatest, in particular least developed countries, African countries, small island developing States and landlocked developing countries, in accordance with their national plans and programmes
	Target 14.5	By 2020, conserve at least 10 per cent of coastal and marine areas, consistent with national and international law and based on the best available scientific information
	Target 15.1	By 2020, ensure the conservation, restoration and sustainable use of terrestrial and inland freshwater ecosystems and their services, in particular forests, wetlands, mountains and drylands, in line with obligations under international agreements
	15.4	By 2030, ensure the conservation of mountain ecosystems, including their biodiversity, in order to enhance their capacity to provide benefits that are essential for sustainable development
	17.6	Enhance North-South, South-South and triangular regional and international cooperation on and access to science, technology and innovation and enhance knowledge-sharing on mutually agreed terms, including through improved coordination among existing mechanisms, in particular at the United Nations level, and through a global technology facilitation mechanism
	17.8	Fully operationalize the technology bank and science, technology and innovation capacity-building mechanism for least developed countries by 2017 and enhance the use of enabling technology, in particular information and communications technology

(2) ***10.6** Ensure enhanced representation and voice for developing countries in decision-making in global international economic and financial institutions in order to deliver more effective, credible, accountable and legitimate institutions*

'unexplained'

**Target 10.6 → ? ←**	Target 3.2	By 2030, end preventable deaths of newborns and children under 5 years of age, with all countries aiming to reduce neonatal mortality to at least as low as 12 per 1,000 live births and under-5 mortality to at least as low as 25 per 1,000 live births
	Target 3.3	By 2030, end the epidemics of AIDS, tuberculosis, malaria and neglected tropical diseases and combat hepatitis, water-borne diseases and other communicable diseases
	Target 3.9	By 2030, substantially reduce the number of deaths and illnesses from hazardous chemicals and air, water and soil pollution and contamination
	Target 3.b	Support the research and development of vaccines and medicines for the communicable and non-communicable diseases that primarily affect developing countries, provide access to affordable essential medicines and vaccines, in accordance with the Doha Declaration on the TRIPS Agreement and Public Health, which affirms the right of developing countries to use to the full the provisions in the Agreement on Trade-Related Aspects of Intellectual Property Rights regarding flexibilities to protect public health, and, in particular, provide access to medicines for all
	Target 5.5	Ensure women's full and effective participation and equal opportunities for leadership at all levels of decision-making in political, economic and public life
	Target 7.1	By 2030, ensure universal access to affordable, reliable and modern energy services
	Target 10.b	Encourage official development assistance and financial flows, including foreign direct investment, to States where the need is greatest, in particular least developed countries, African countries, small island developing States and landlocked developing countries, in accordance with their national plans and programmes

	Target 14.5	By 2020, conserve at least 10 per cent of coastal and marine areas, consistent with national and international law and based on the best available scientific information
	Target 15.1	By 2020, ensure the conservation, restoration and sustainable use of terrestrial and inland freshwater ecosystems and their services, in particular forests, wetlands, mountains and drylands, in line with obligations under international agreements
	Target 15.4	By 2030, ensure the conservation of mountain ecosystems, including their biodiversity, in order to enhance their capacity to provide benefits that are essential for sustainable development
	Target 17.6	Enhance North-South, South-South and triangular regional and international cooperation on and access to science, technology and innovation and enhance knowledge-sharing on mutually agreed terms, including through improved coordination among existing mechanisms, in particular at the United Nations level, and through a global technology facilitation mechanism
	Target 17.8	Fully operationalize the technology bank and science, technology and innovation capacity-building mechanism for least developed countries by 2017 and enhance the use of enabling technology, in particular information and communications technology

(3) ***15.5*** *Take urgent and significant action to reduce the degradation of natural habitats, halt the loss of biodiversity and, by 2020, protect and prevent the extinction of threatened species*

'unexplained'

**Target 15.5 → ? ←**	Target 3.1	By 2030, reduce the global maternal mortality ratio to less than 70 per 100,000 live births
	Target 3.2	By 2030, end preventable deaths of newborns and children under 5 years of age, with all countries aiming to reduce neonatal mortality to at least as low as 12 per 1,000 live births and under-5 mortality to at least as low as 25 per 1,000 live births
	Target 3.9	By 2030, substantially reduce the number of deaths and illnesses from hazardous chemicals and air, water and soil pollution and contamination

	Target 3.d	Strengthen the capacity of all countries, in particular developing countries, for early warning, risk reduction and management of national and global health risks
	Target 6.2	By 2030, achieve access to adequate and equitable sanitation and hygiene for all and end open defecation, paying special attention to the needs of women and girls and those in vulnerable situations
	Target 7.1	By 2030, ensure universal access to affordable, reliable and modern energy services
	Target 14.5	By 2020, conserve at least 10 per cent of coastal and marine areas, consistent with national and international law and based on the best available scientific information
	Target 15.1	By 2020, ensure the conservation, restoration and sustainable use of terrestrial and inland freshwater ecosystems and their services, in particular forests, wetlands, mountains and drylands, in line with obligations under international agreements
	Target 15.4	By 2030, ensure the conservation of mountain ecosystems, including their biodiversity, in order to enhance their capacity to provide benefits that are essential for sustainable development
	Target 17.8	Fully operationalize the technology bank and science, technology and innovation capacity-building mechanism for least developed countries by 2017 and enhance the use of enabling technology, in particular information and communications technology

Table S4. Percent increases (a) and decreases (b) in influence of the SDG targets in the SDG systems model compared to percent changes in influence using different weighting schemes. Three models were run as a sensitivity analyses to test the effects of the applied weighting scheme (25 for added connections). All SDG targets that either increase (a) or decrease (b) compared to the initial influence on the final factor of a sustainable world are shown. “N/A” indicates no change in target influence. The percent increase (and percent decrease) of SDG targets represents their change in influence on

the objective a sustainable world due to the connections among targets. The five greatest increases are shown in bold with rankings 1-5 (a). SDGs 11 and 12 have no extracted targets for the SDG system model.

Percent increase (lever targets)

SDG	Target	SDG Systems Model	1) Relative Weight	2) 50 Weight	3) 10 Weight
SDG1	1.5	56	18	125	21
SDG2	2.1	76	50	338	22
SDG3	3.1	122	131	806	21
	3.2	81	102	625	14
	3.3	39	43	244	2
	3.7	34	37	138	11
	3.9	25	26	162	7
	3.b	12	N/A	12	N/A
	3.c	181	216	969	41
	3.d	117	133	556	33
SDG4	4.2	43	40	306	5
SDG5	5.5	**418 (2)**	**441 (3)**	**2169 (2)**	**101 (2)**
SDG6	6.1	76	50	338	22
	6.2	142	92	688	37
	6.6	19	15	119	2
SDG7	7.1	**272 (5)**	104	**1569 (5)**	**57 (4)**
	7.3	28	20	150	4
SDG8	8.a	220	62	1562	30
SDG9	9.4	25	40	50	50
	9.c	206	142	938	51
SDG10	10.b	64	65	375	20
SDG13	13.1	25	34	50	10
SDG14	14.5	92	120	744	10
SDG15	15.1	204	**222 (5)**	1012	51
	15.2	30	29	162	4
	15.4	**275 (4)**	**304 (4)**	**1687 (4)**	**51 (5)**
	15.a	42	42	144	17
	15.b	14	14	69	2
SDG17	17.6	**384 (3)**	**709 (2)**	**1981 (3)**	**92 (3)**
	17.8	**480 (1)**	**823 (1)**	**2231 (1)**	**125 (1)**

a) **Percent decrease (hurdle targets)**

SDG	Target	SDG Systems Model	1) Relative Weight	2) 50 Weight	3) 10 Weight
SDG3	3.b	N/A	-22	N/A	-4
SDG10	10.6	228	-222	-1631	-18
	10.b	N/A	-135	N/A	N/A
SDG15	15.5	163	-178	-900	-21
SDG16	16.8	218	-286	-1275	-20
